# Effect of Polyelectrolyte Charge Density on the Linear Viscoelastic Behavior and Processing of Complex Coacervate Adhesives

**DOI:** 10.1021/acs.macromol.3c02352

**Published:** 2024-01-01

**Authors:** Larissa van Westerveld, Théophile Pelras, Anton H. Hofman, Katja Loos, Marleen Kamperman, Julien Es Sayed

**Affiliations:** † Polymer Science, Zernike Institute for Advanced Materials, University of Groningen, Nijenborgh 4, Groningen 9747 AG, The Netherlands; ‡ Macromolecular Chemistry and New Polymeric Materials, Zernike Institute for Advanced Materials, University of Groningen, Nijenborgh 4, Groningen 9747 AG, The Netherlands

## Abstract

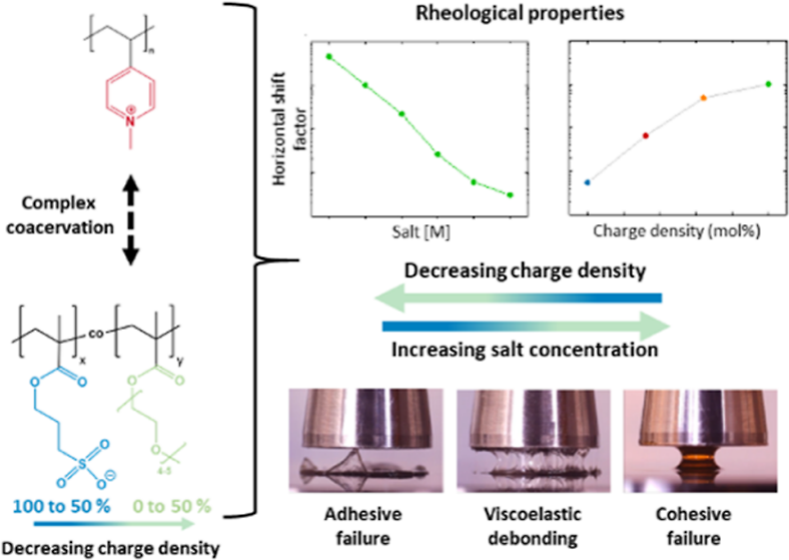

It is well-known that the phase behavior and physicochemical and adhesive properties of complex coacervates are readily tuneable with the salt concentration of the medium. For toxicity reasons, however, the maximum applicable salt concentration in biomedical applications is typically low. Consequently, other strategies must be implemented in order to optimize the properties of the resulting complex coacervates. In this work, the effect of the charge density of a strong polyanion on the properties of complex coacervates was studied. To control this charge density, statistical anionic/charge-neutral hydrophilic copolymers were synthesized by means of an elegant protection/deprotection strategy and subsequently complexed with a strong polycation. The resulting complexes were observed to have an increasing water content as well as faster relaxation dynamics, with either increasing salt concentration or decreasing charge density. Time–salt and time–salt–charge density superpositions could be performed and showed that the relaxation mechanism of the complex coacervates remained unchanged. When the charge density was decreased, lower salt concentration complexes became suitable for viscoelastic adhesion with improved injectability. Such complex coacervates are promising candidates for injectable biomedical adhesives.

## 1 Introduction

More than a decade ago, it was revealed that aquatic organisms, such as sandcastle worms and mussels, are able to secrete an initially aqueous fluid mixture of charged proteins that later hardens to create strong bonding with various hard surfaces under marine conditions.^1,2^ It has further been highlighted that several properties of these formulations are shared with those of materials known as (complex) coacervates. Complex coacervates are water-based materials that are formed through electrostatic interactions between oppositely charged polyelectrolytes.^3,4^ Since then, several groups have focused on designing synthetic polymer-based complex coacervates that can be used as injectable wet adhesives.^5−8^ From a physical standpoint, the adhesive properties of complex coacervates mainly rely on their viscoelastic nature, similar to conventional dry pressure-sensitive adhesives (PSAs).^9−12^ In fact, to obtain an injectable complex coacervate adhesive, an optimal balance has to be found between viscous and elastic behavior. The fluid-like viscous behavior affords injectability, spreading on the surface that it needs to attach to, and bulk energy dissipation during debonding, and the solid-like elastic behavior affords load-bearing and prevents unwanted creep. It has been widely reported that the viscoelastic properties of complex coacervates, and consequently the wet adhesive performance, can be easily controlled via the salt concentration at which it is prepared.^6−8,13−15^ As a general trend, a complex coacervate made at relatively high salt concentrations behaves as a viscous fluid that can flow easily, while at relatively low salt concentrations, it tends to behave like a solid material. At the molecular scale, salt acts as a dopant that increases the dissociation rate of the electrostatic interactions between oppositely charged polyelectrolytes. This results in an increase in the water content and a decrease in the viscosity of the material.^16^ Even though injectable adhesives are becoming an enormous asset for biomedical applications, high salt concentrations (i.e., far above physiological concentrations) are not always preferred due to increased toxicity.^17^ As a consequence, alternatives have to be found to tune and optimize the viscoelastic properties, injectability, and wet adhesive properties, of complex coacervates within a narrow range of salt concentrations.

One promising approach to reach this goal is to chemically encode constitutive polymers with predefined molecular characteristics. More specifically, the inclusion of a controlled number of uncharged but functional units within the polyelectrolyte backbone has been shown to greatly influence the viscoelasticity and adhesive performance of the formulated complex coacervates. Reactive units such as catechols,^1,18^ photoreactive moieties,^19^ or ligands^20^ are then often regarded as stickers that associate and slow down the dynamics within the material.^21^ This latter approach was revealed to be very efficient in transitioning the complex coacervates from a viscoelastic liquid into an elastic solid, thus enhancing the adhesion performance after an adapted post-treatment (curing with pH, light, and addition of transition metal). Recently, by including self-associative hydrophobic isobutyl units within the backbone of one of the polyelectrolytes, we demonstrated that the best wet adhesion performance of the complex coacervates was shifted to higher salt concentrations compared to the unmodified system.^22^ Concomitantly, this was accompanied by a decrease in the water content and delayed relaxation dynamics that could hamper their injectability. From this study, the following questions arose: (i) how can the effect of the increased association between chains through hydrophobic interactions be decorrelated from the effect of the dilution of the charges along the chains on the complex coacervate physical–chemical properties? (ii) At relatively low salt concentrations, can we reconcile the trade-off between injectability and good wet adhesion by diluting the charges along the polyelectrolyte chains?

Building on these results, we hypothesize that replacing these charge-neutral hydrophobic associative units with nonassociative charge-neutral hydrophilic units would help to answer these questions. Indeed, it has previously been reported that incorporation of charge-neutral hydrophilic groups decreases the salt resistance and simultaneously speeds up the relaxation dynamics.^23−26^ However, studies in relation to the adhesive properties and injectability of such complex coacervates have never been performed.

**Scheme 1:**
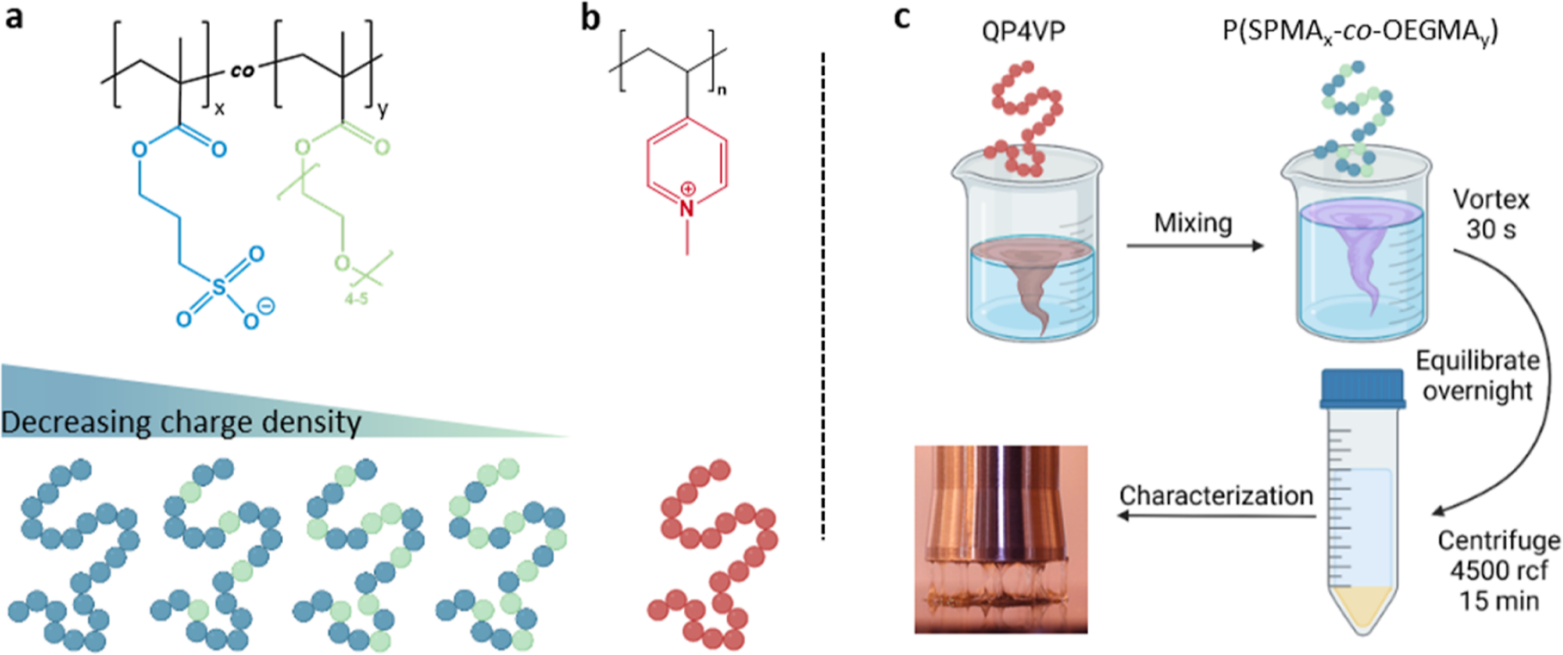
Molecular Structures and Schematic Representation of (a) P(SPMA_*x*_-*co*-OEGMA_*y*_) with Decreasing Charge Density *x* (i.e., SPMA Repeat Units) from Left to Right and (b) QP4VP; (c) Schematic Representation of the Preparation Procedure of the P(SPMA_*x*_-*co*-OEGMA_*y*_)/QP4VP Complex Coacervates

In this work, we introduced increasing amounts of oligo([ethylene glycol] methyl ether methacrylate) (OEGMA) units along the backbone of poly(3-sulfopropyl methacrylate) (PSPMA) as hydrophilic spacers to control the charge density of the resulting strong polyanion poly[(3-sulfopropyl methacrylate)-*co*-(oligoethylene glycol)] methyl ether methacrylate) sodium salt, P(SPMA_*x*_-*co*-OEGMA_*y*_) (Scheme 1). Poly(ethylene glycol) is known for its strong hydration layers and biocompatibility, which have pushed its implementation in biomedicine and antifouling coatings.^27−32^

To this end, OEGMA and 3-isobutoxysulfopropyl methacrylate (BSPMA), a precursor to SPMA, were first copolymerized in different ratios using reversible addition–fragmentation chain transfer (RAFT) polymerization, ranging from no OEGMA up to 50 mol % OEGMA. Subsequent deprotection of BSPMA’s sulfonate moiety led to the desired P(SPMA_*x*_-*co*-OEGMA_*y*_) copolyelectrolytes. Complex coacervates were obtained by combining these copolymers with poly(*N*-methyl-4-vinylpyridinium iodide) (QP4VP), which is a strong polycation. PSPMA and QP4VP were chosen for their strong polyelectrolyte character (i.e., charge density independent of the pH) and to closely match our previous study on a hydrophobic coacervate system.^22^ OEGMA (300 g/mol) was selected for its charge-neutral character yet excellent solubility and its molecular weight closely matching that of BSPMA.^33^ The effect of the OEGMA content (i.e., charge density) of the polyanion on the phase behavior and viscoelasticity of the resulting complex coacervates was investigated. Finally, the injectability and adhesion performance of these different formulations were assessed via probe-tack measurements.

## 2 Experimental Section

### 2.1 Materials

BSPMA was synthesized according to a previously reported literature procedure.^22,34,35^ Anhydrous *N*,*N*-dimethylformamide (DMF; 99.8%), poly(4-vinylpyridine) (P4VP; *M*_w_ = 60,000 g mol^–1^), iodomethane (MeI; 99.0%), deuterated solvents, the RAFT agent 4-cyano-4-(thiobenzoylthio)pentanoic acid (CTBPA), and sodium chloride (NaCl) were acquired from Sigma-Aldrich. Oligo(ethylene glycol) methyl ether methacrylate (OEGMA; *M*_n_ = 300 g mol^–1^) was obtained from Sigma-Aldrich and passed through a short activated basic alumina column prior to polymerization. Sodium iodide (NaI) (99.5%, anhydrous) was obtained from Acros-Organics. Azobisisobutyronitrile (AIBN) was obtained from Sigma-Aldrich and recrystallized twice from methanol. All other analytical grade solvents were obtained from Boom. Water was of R.O. quality (10 μS cm^–1^, equivalent to 10^–4^ M NaCl).

### 2.2 Characterization

#### 2.2.1 Proton Nuclear Magnetic Resonance (^1^H NMR)

^1^H NMR spectra were obtained with either a Varian VXR-400 or an Agilent 400 MR spectrometer at 25 °C. D_2_O (99.9%), DMSO-*d*_6_ (99.5%) and CDCl_3_ (99.8%) were used depending on the solubility of the respective polymers. Chemical shifts (δ) are shown relative to the residual solvent peak in parts per million (ppm). *M*_n,NMR_ is the calculated molecular weight based on the monomer conversion (^1^H NMR) and the theoretical maximum molecular weight (monomer/CTA ratio).

#### 2.2.2 Size Exclusion Chromatography

A Viscotek GPCmax system equipped with a TDA 302 triple detector array (refractive index, viscosity, and light scattering) and two analytical columns (Agilent Technologies PolarGel-M and PolarGel-L, 30 cm × 8 μm) was used to perform SEC. DMF containing 0.01 M LiBr was used as the eluent at a flow rate of 1.0 mL min^–1^, and both columns as well as the detectors were held at 50 °C. Near-monodisperse poly(methyl methacrylate) (PMMA) standards (Polymer Standard Devices) were used for calibration, and the samples were filtered over a 0.2 μm PTFE filter prior to injection. Conventional calibration on Viscotec OmniSEC software was used to determine the molecular weights of the homopolymers (Table S1).

#### 2.2.3 Water Content Measurement

The complex coacervate samples were dried in an oven to determine the water content. The samples were made in a total volume of 1.6 mL. The supernatant was first removed by decanting or pipetting, and the remaining drops were carefully removed with tissue paper. The complex coacervate phases were dried in an oven at 120 °C. The total weight of material (typically between 50 and 100 mg depending on the salt amount) was monitored over a period of 4 h and was found to become stable after 1 h. The relative weight after drying was attributed to the full evaporation of water; the weight loss was therefore considered to be the water content. All measurements were performed in triplicate.

#### 2.2.4 Linear Shear Rheology

A strain-controlled Anton Paar MCR302e rheometer was used to perform linear viscoelastic measurements on the complex coacervates. Strain sweeps from 0.10 to 10% at a fixed frequency of 100 rad s^–1^ were performed to determine the linear viscoelastic regime of each sample. Then, frequency sweeps were performed from 100 to 0.10 rad s^–1^ at a fixed strain of 1%. All samples were measured with cone–plate geometry, where the cone had a diameter of 25 mm and a 1° angle (CP25-1). The samples were allowed to relax until the normal force was less than 1 N before starting the measurement. 2 mL of the supernatant was deposited around the geometry to prevent the samples from drying out during the experiments. Furthermore, a minimum cutoff torque of 0.001 mN·m was set for all measurements.

#### 2.2.5 Time–Salt (–Charge Density) Superposition of the Rheological Data

The applicability of the time–salt superposition (TSS) for the samples prepared with polyanions with different charge densities was verified via a Van Gurp–Palmen representation (phase shift δ as a function of the complex modulus G*). This is a frequency-independent plot, which is powerful for checking rheological complexities in polymeric systems.^36−38^ After this check, the horizontal shift factors (*a*_s_) were determined by shifting the loss factor curves over a single master curve. For each charge density series, the sample prepared at 0.25 M NaCl was used as the reference. Finally, the vertical shift factor (*b*_s_) was obtained by shifting the moduli curves with respect to the reference.

Time–salt–charge density superposition (TSCDS) was again verified with a Van Gurp–Palmen plot. The horizontal shift factor (*a*_CD_) was determined by shifting the loss factor curves over a single master curve. The curves for different charge densities were shifted with respect to those of the cc100 series. The vertical shift factor (*b*_CD_) was then determined by shifting the curves vertically to overlap with the reference.

#### 2.2.6 Probe-Tack Experiments

The probe-tack measurements were performed on an Anton Paar MCR302e rheometer in the “Tack, penetration” mode, inspired by the procedure reported by Vahdati et al.^7^ The method is the same as the one we followed in a previous study wherein the procedure is extensively explained.^22^ Briefly, around 0.3 mL of each sample was loaded either with a spatula (for the more solid samples) or with a positive displacement syringe (for the most liquid samples) on the stainless-steel bottom plate of the rheometer. The top plate (10 mm diameter stainless-steel sandblasted parallel plate, PP10/S) was lowered to an initial gap *h*_0_ = 150 μm. Stainless-steel plates were chosen as a model system to prevent any contribution to the work of adhesion from the deformation of a soft substrate or the penetration of the adhesive into the substrate. A contact time of 1 min was respected, and the probe was subsequently retracted at a speed of *v* = 100 μm/s (nominal strain rate is 0.67 s^–1^). The normal force (*F*_N_) was recorded as a function of displacement (*h*). Six replicates were performed for each sample. Between each measurement, the top and bottom surfaces were thoroughly washed with deionized water and ethanol. Stress–strain curves were obtained by converting the displacement into strain (ε) using eq 1 and converting the normal force into stress (σ) using eq 2.
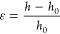
1
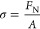
2where *A* is the area of the probe.

The work of adhesion (*W*_adh_) was calculated from the integration of the stress–strain curve by using eq 3.
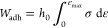
3

The maximum strain was set when the normal force reached zero (±0.06 N).

### 2.3 Polymer Synthesis

#### 2.3.1 Synthesis of Poly(3-isobutoxysulfopropyl Methacrylate)-*co*-(oligo[ethylene Glycol] Methyl Ether Methacrylate) P(BSPMA_*x*_-*co*-OEGMA_*y*_)

P(BSPMA_*x*_-*co*-OEGMA_*y*_) was synthesized by RAFT polymerization (Table 1). An example of the 50/50 ratio copolymer is given below. For this polymer, 1.81 g of BSPMA (6.85 mmol; 250 equiv) and 2.05 g of OEGMA (6.85 mmol; 250 equiv) were charged in a round-bottom flask equipped with a stirring egg. Then, 6.0 mg of CTBPA (0.0274 mmol; 1 equiv) and 0.45 mg of AIBN (2.74 × 10^–3^ mmol; 0.13 equiv) were obtained from stock solutions of 14.7 and 5.38 mg mL^–1^ in DMF, respectively. Then, 4.5 mL of DMF was added, and an aliquot was withdrawn for ^1^H NMR conversion analysis before the solution was sparged with argon for 15 min. When the argon flow was closed, the reaction mixture was immersed in a preheated oil bath at 70 °C and stirred overnight. After 17 h, the polymerization was stopped by removing the sample from the oil bath, cooling to room temperature, and opening to air before withdrawal of an aliquot for ^1^H NMR conversion analysis. Before precipitation, it was diluted with approximately 5 mL of acetone and precipitated into 6:1 *n*-hexane/ethanol. The obtained sticky pink product was redissolved in minimal THF and precipitated twice into *n*-hexane. Finally, the product was redissolved in acetone, transferred to a clean vial, and dried in a 60 °C oven overnight. 3.61 g of a pink viscous material was obtained (93% yield). For all three copolymers, ^1^H NMR and SEC samples were analyzed to determine the monomer conversions and the lengths of the polymer chains (Figures S3–S5 and Table S1).

**Table 1:** Characteristics of the P(SPMA_*x*_-*co*-OEGMA_*y*_) Copolymers Used in This Study

sample	DP_total_^a^	DP_SPMA_^a^	DP_OEGMA_^a^	*M*_n NMR_ (kg mol^–^^1^)^a^	*M*_n SEC_ (kg mol^–^^1^)^a^	*D̵*^b^
SPMA100	514	514	0	136	122	1.36
SPMA82	405	332	73	110	128	1.43
SPMA66	420	277	143	116	149	1.57
SPMA50	430	215	215	114	152	1.73

a Determined by ^1^H NMR conversion samples.

b Determined by SEC in DMF with 0.01 M LiBr on the protected intermediates and calibrated against near-monodisperse PMMA standards; the relative compositions were determined by ^1^H NMR on the purified products.

#### 2.3.2 Synthesis of Poly[(3-sulfopropyl Methacrylate)-*co*-(oligoethylene Glycol Methyl Ether Methacrylate] Sodium Salt P(SPMA_*x*_-*co*-OEGMA_*y*_)

The PBSPMA homopolymer and P(BSPMA_*x*_-*co*-OEGMA_*y*_) copolymers were deprotected following a previously reported procedure.^22,34,35^ Briefly, the polymers were dissolved in DMSO (50 mg mL^–1^), 3 equiv of NaI respective to the BSPMA units was added, and the solution was heated to 70 °C for 20 h. Polymers were subsequently precipitated in 1:2 pentane/ethanol and washed with 2:1 pentane/ethanol and pentane. The P(SPMA_*x*_-*co*-OEGMA_*y*_) chains were redissolved in water and freeze-dried (Figures S4 and S5 and Table S2). Despite our best efforts, iodine byproducts always stuck to OEGMA, hence the increasingly intense orange/red color with higher OEGMA ratios. The obtained polymers are further named following the mol % of SPMA units: SPMA100, SPMA82, SPMA66, and SPMA50.

#### 2.3.3 Synthesis of Poly(*N*-methyl-4-vinyl Pyridinium Iodide) (QP4VP)

P4VP was quaternized according to an adapted procedure from Sadman and co-workers.^39^ To obtain QP4VP, 4.00 g (38 mmol) of P4VP was dissolved in 40 mL of DMSO before 10.8 g (76 mmol, 2 equiv) of MeI was added dropwise, and the reaction was stirred for 4 h at room temperature. Excess MeI was first removed by bubbling the solution with nitrogen before precipitation was performed in acetone, and the polymer was redissolved in water. QP4VP was retrieved after freeze-drying and with high yield, i.e., quaternization ≥99%, 9.06 g (yield 96%) (Figure S6).

### 2.4 Complex Coacervate Preparation

Polymer stock solutions were prepared by dissolving PSPMA or P(SPMA_*x*_-*co*-OEGMA_*y*_) polyanions and QP4VP polycation separately in water at a concentration of 0.20 M charged monomers. A stock solution of 5.0 M NaCl was used. The salt concentrations investigated for the complex coacervate samples were varied from 0.00 to 1.25 M in increments of 0.25 M. To prepare a complex coacervate, first the correct amounts of water and NaCl solution were mixed together, then QP4VP stock solution was added, and last PSPMA or P(SPMA_*x*_-*co*-OEGMA_*y*_) was added. Finally, the mixture was vortexed for 30 s, and the sample was left to equilibrate overnight. For complexation, the total charged monomer concentration was set to 0.10 M with a positive-to-negative charge ratio of 1 to 1, i.e., full charge compensation. Therefore, higher OEGMA fractions resulted in a higher copolymer/QP4VP weight ratio (Table S3 for the complete calculation). The next morning, the mixtures were centrifuged for 15 min at 4500 rpm to collect the complex coacervate phase at the bottom of the tube. Complex coacervates prepared with different SPMA/OEGMA ratios were named according to the mol % of charged SPMA units: cc100, cc82, cc66, and cc50, where cc stands for “complex coacervate”.

## 3 Results and Discussion

### 3.1 Polymer Synthesis

**Figure 1:**
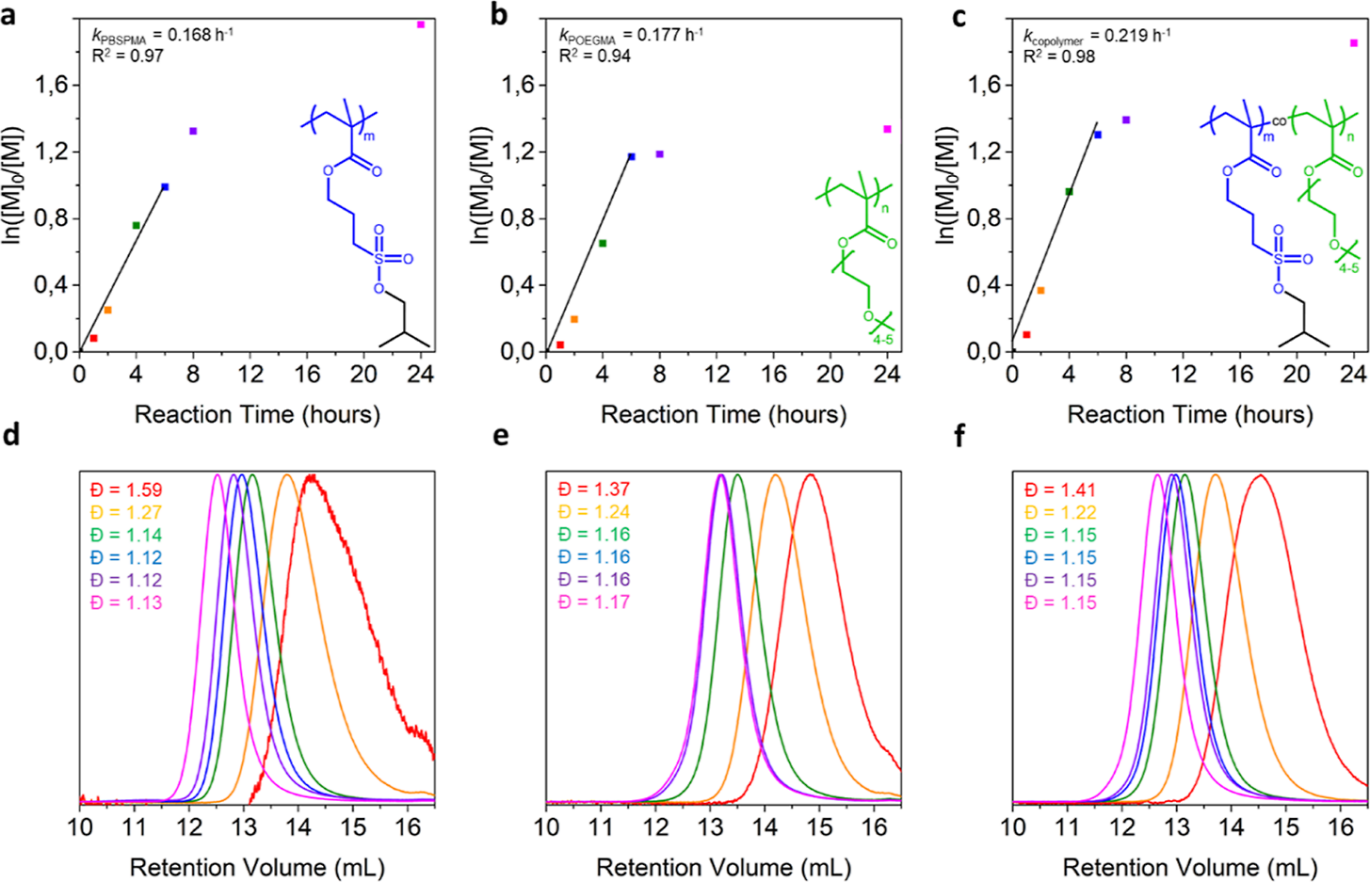
Kinetic studies on (a) BSPMA and (b) OEGMA homopolymerization and (c) 50/50 BSPMA/OEGMA copolymerization, extracted from ^1^H NMR conversion data. (d–f) Corresponding SEC chromatograms measured in DMF with 0.01 M LiBr.

The PBSPMA homopolymer and P(BSPMA_*x*_-*co*-OEGMA_*y*_) copolymers were synthesized using a two-step procedure (i.e., RAFT polymerization followed by nucleophilic deprotection). While the production of the strong anionic homopolymer is straightforward, the feasibility of a proper statistical copolymerization had to be investigated.^40,41^ The homopolymerization kinetics of either BSPMA or OEGMA (∼100 equiv to the RAFT agent) were followed using a combination of ^1^H NMR spectroscopy and SEC (Figure 1). The ln([M]_0_/[M]) vs time plots, constructed from ^1^H NMR conversion data, indicate very similar kinetic profiles between the two methacrylates with comparable apparent rate constants of *k*_BSPMA_ = 0.168 h^–1^ and *k*_OEGMA_ = 0.177 h^–1^. SEC chromatograms also evidenced the absence of tailing or chain–chain termination, accompanied by a reduction in the dispersity over time, with final values *D̵* < 1.2, indicating good control over the polymerization. To further verify that both monomers would be included in the polymer at the same rate (i.e., to obtain true statistical copolymers in lieu of gradients), another kinetic study was performed on the copolymerization of BSPMA and OEGMA. For each monomer, ∼50 equiv of the RAFT agent was introduced into the flask, and the reaction was performed in a similar fashion. Not only were small aliquots of the reaction mixture withdrawn for ^1^H NMR conversion and SEC analyses, but larger aliquots were also taken to isolate material for thorough ^1^H NMR analysis (Figure S1). While several proton signals overlap in the 3.5–4.2 ppm region, signals e (BSPMA CH_2_, 2H, 3.21 ppm), j (OEGMA CH_3_, 3H, 3.38 ppm), and i″ (OEGMA CH_2_ before the ether bond, 2H, 3.55 ppm) can be used to determine the relative molar fractions of the repeating units. This enabled the verification that the e/j/i″ signal ratios remained at 2:3:2 across the polymerization kinetics, confirming the successful statistical copolymerization. Importantly, the protective groups of BSPMA units remained unaltered during the polymerization, enabling the synthesis of precursors that are readily soluble in organic solvents and hence allow for more straightforward analysis.

**Figure 2:**
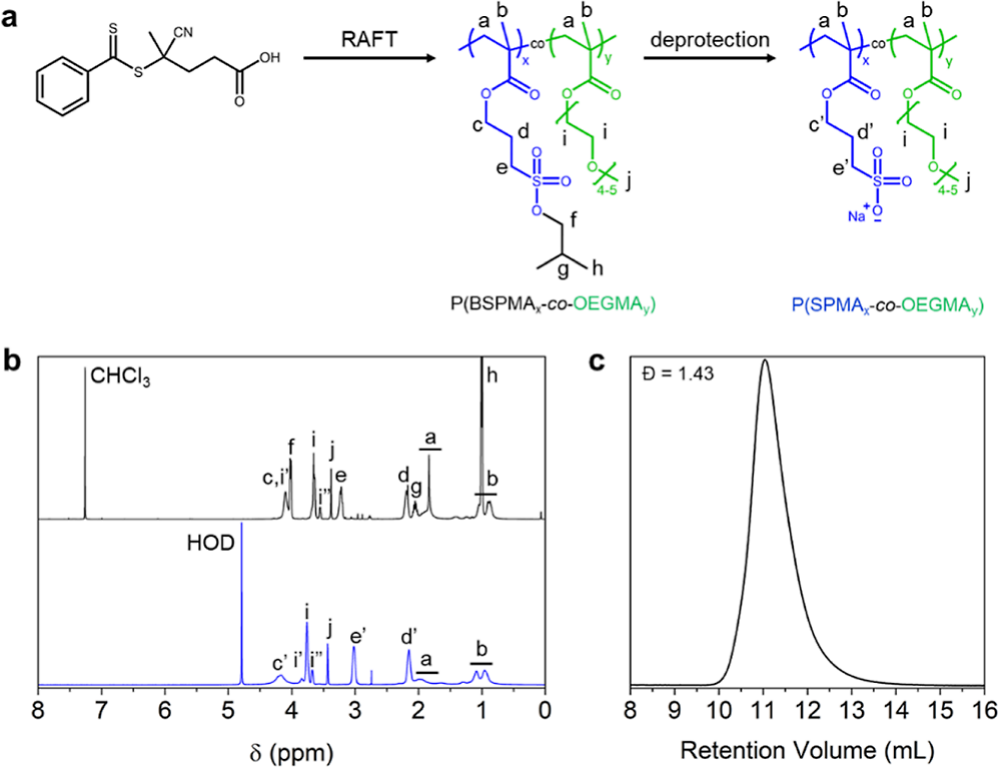
Synthesis and characterization of a P(SPMA_*x*_-co-OEGMA_*y*_) copolymer. (a) Two-step synthesis route to produce the polymer via a protected P(BSPMA_*x*_-*co*-OEGMA_*y*_) intermediate. (b) ^1^H NMR spectra of protected P(BSPMA_82_-*co*-OEGMA_18_) (black, CDCl_3_) and deprotected P(SPMA_82_-*co*-OEGMA_18_) (blue, D_2_O). (c) SEC elugram of the protected P(BSPMA_82_-*co*-OEGMA_18_) intermediate was measured in DMF with 0.01 M LiBr.

Next, a range of P(BSPMA_*x*_-*co*-OEGMA_*y*_) copolymers, with BSPMA molar fractions ranging from *x*_BSPMA_ = 0.82–0.50, were produced in a similar fashion. A total degree of polymerization of DP ∼ 420 was kept across the series to circumvent the possible effects of chain length on the complex coacervates. ^1^H NMR and SEC (Figures 2 and S2) were used to verify the chemical composition of the samples and the absence of chain–chain coupling, respectively. Then, removal of the isobutoxy protective groups was performed with 3 equiv sodium iodide in DMSO and stirring at 70 °C for 20 h,^34,35^ yielding a series of P(SPMA_*x*_-*co*-OEGMA_*y*_) anionic/charge-neutral hydrophilic copolymers. To ensure that only BSPMA units would be subject to nucleophilic deprotection, a “negative control” reaction was also performed on a POEGMA homopolymer, and the ^1^H NMR spectrum and SEC elugram were compared to those of the pristine sample (Figure S3). The successful deprotection of the copolymers, now readily soluble in D_2_O, was confirmed by ^1^H NMR with the loss of isobutoxy signals (CH_3_, 6H, 1.00 ppm; CH_2_, 2H, 4.02 ppm; CH, 1H, 2.05 ppm) (Figures S4 and S5). The characteristics of the P(SPMA_*x*_-*co*-OEGMA_*y*_) copolymers produced in this study are summarized in Table 1. The marginally higher dispersities of the copolymers with a higher ratio of OEGMA may result in a broadening of the distribution of relaxation times. However, this effect is relatively limited for complex coacervates with no chain entanglements, where the longest characteristic relaxation time is mainly dictated by the lifetime of electrostatic interactions.^22,42^

Finally, commercially available poly(4-vinylpyridine), P4VP, was quaternized using iodomethane in DMSO, following a previously reported procedure (Figure S6).^39^ This enabled the production of the positively charged polymer poly(*N*-methyl-4-vinyl pyridinium iodide), QP4VP, required for the formation of the complex coacervates. Note that the presence of permanent charges within the anionic/charge-neutral and cationic polyelectrolytes ensures efficient electrostatic complexation, with no influence of the pH of the medium.^43^

### 3.2 Phase Behavior of the P(SPMA_*x*_-*co*-OEGMA_*y*_)/QP4VP Complex Coacervates

**Figure 3:**
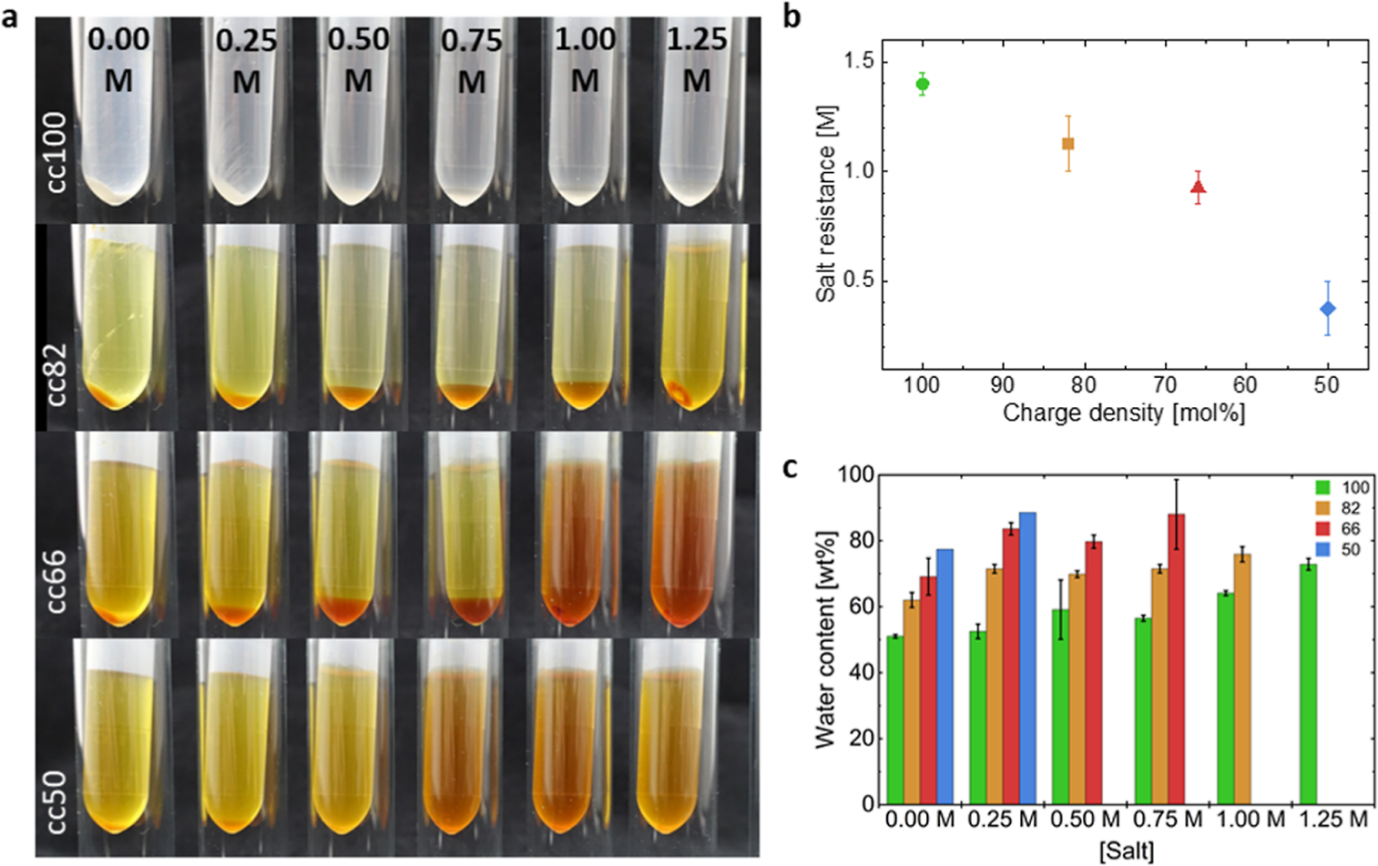
(a) Photographs of the complex coacervate series with a decreasing charge density of the P(SPMA_*x*_-*co*-OEGMA_*y*_) polyanion from top to bottom from 0.00 to 1.25 M NaCl. (b) Evolution of the salt resistance of the complex coacervates as a function of the charge density of the P(SPMA_*x*_-*co*-OEGMA_*y*_) polyanion. (c) Water content (wt %) of the complex coacervates at different salt concentrations and with varying charge density of the P(SPMA_*x*_-*co*-OEGMA_*y*_) polyanion.

Complex coacervates were formed at a 1:1 charge ratio between the negative charges borne by the P(SPMA_*x*_-*co*-OEGMA_*y*_) copolymers (SPMA100, SPMA82, SPMA66, or SPMA50) and the positive charges borne by the QP4VP chains (i.e., full charge compensation) and at a fixed total charge concentration of 0.10 M (Scheme 1 and Table S3). The four series of complex coacervates were named according to the mol % of charged SPMA in the copolymers: cc100, cc82, cc66, and cc50, where cc stands for “complex coacervate”. The salt concentration was varied between 0.00 and 1.25 M NaCl with steps of 0.25 M (Figure 3a). The salt resistance (i.e., the salt concentration above which no phase separation is visually observed) for the four systems was first determined and is reported in Figure 3b. We observed a large difference in salt resistance between cc100 and cc50 of more than 1 M with values of 1.40 and 0.375 M, respectively, indicating a strong dependence of the charge density of the P(SPMA_*x*_-*co*-OEGMA_*y*_) copolymers on the driving force for complex coacervation. The following trend is generally observed: the higher the OEGMA neutral hydrophilic content in the polyanions, the lower the salt resistance. Note that the orange hue observed in coacervates produced from high OEGMA-containing copolymers originates from the oxidation of excess sodium iodide into iodine during the deprotection. Iodine and poly(ethylene glycol) have a certain affinity, which cannot easily be overcome by careful wash cycles.^44^ Then, the water content of the corresponding complex coacervates was measured and is reported in Figure 3c. For all four series with a given charge density of the polyanion, the water content is shown to increase with the added salt concentration. As an example, for the cc100 series, the water content increases from 51 wt % at 0.00 M NaCl to 72 wt % at 1.25 M NaCl, while for the most extreme series, cc50, it also increases from 78 wt % at 0.00 M NaCl to 84 wt % at 0.25 M NaCl. As extensively reported for complex coacervates, salt acts as an efficient additive to loosen the electrostatic interactions between the oppositely charged polyelectrolytes, which leads to an increasing water content in the obtained materials.^45,46^ Much less investigated in the literature is the effect of the charge density of one of the polyelectrolytes on the water content. In Figure 3c, we also observed that gradually decreasing the SPMA molar ratio from 100 to 50% along the P(SPMA_*x*_-*co*-OEGMA_*y*_) copolymer led to an increase in the water content from 51 to 78 wt % at 0.00 M NaCl and even from 53 to 84 wt % at 0.25 M NaCl. This general trend was observed irrespective of the salt concentration investigated. As a consequence, decreasing the charge density of the polyanion by 50% (transitioning from cc100 to cc50), while keeping the salt concentration relatively low (0.00 or 0.25 M NaCl), was revealed to be as efficient in plasticizing the material with water as increasing the salt concentrations up to 1.25 M NaCl for the system composed of the fully charged polyanion (cc100).

Our results relate to a set of studies performed on complex coacervate systems made with a broad range of polyelectrolyte pairs with varying charge densities.^23−26^ As a few examples, one can refer to the work of Dautzenberg et al., where poly(diallyldimethylammonium chloride) (PDADMAC), a strong polycation, was obtained with decreasing charge density after copolymerization of DADMAC with a water-soluble comonomer (*N*-methyl-*N*-vinylacetamide), NVMA, and subsequently complexed with sodium polystyrenesulfonate, PSSNa.^23^ The resulting complexes transitioned from polymer dense granules to highly swollen particles when increasing the NVMA content from 0 to 74 mol %. More recently, Huang et al. and Neitzel et al. broadened the library of available polymers by performing postmodification of an originally charge-neutral polymer.^25,26^ Consequently, polyelectrolytes (with weak or strong positive or negative charges) were obtained with a controlled amount of either hydrophilic or hydrophobic charge-neutral units. Irrespective of the nature of the charges (weak or strong) and of the chemical nature of the charge-neutral units (hydrophilic or hydrophobic), the authors observed that the salt resistance of the obtained complex coacervates was also decreased with increasing the number of charge-neutral units.

Altogether, these results point to a general trend of a decrease in the driving force for complexation when the charge density of one or both of the polyelectrolytes is reduced.^3,26^ The entropy gain from the released counterions, which is one of the main components for complex coacervation, is actually lowered for a chain that has fewer charges and thus fewer counterions.

### 3.3 Linear Viscoelasticity of the P(SPMA_*x*_-*co*-OEGMA_*y*_)/QP4VP Complex Coacervates

**Figure 4:**
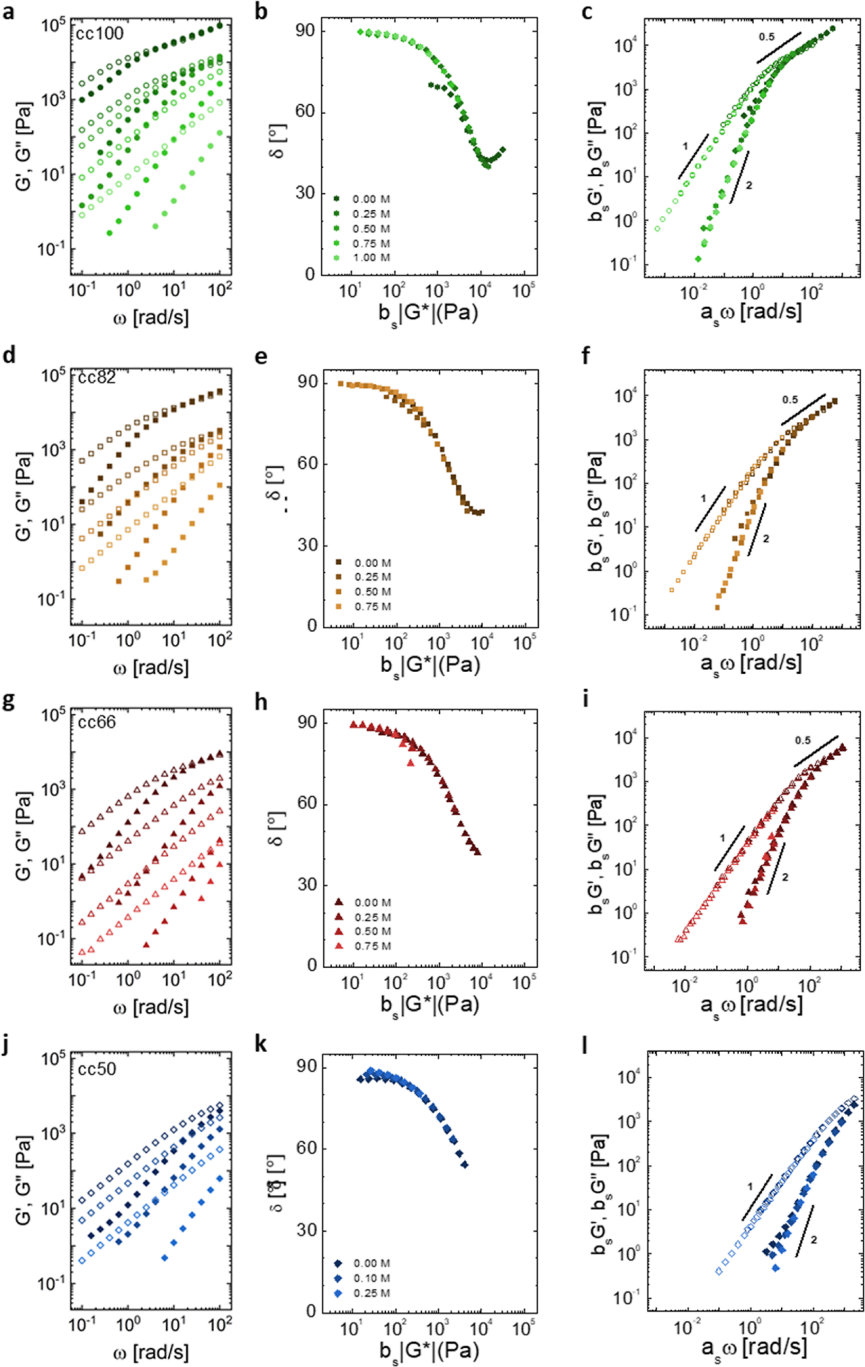
Frequency sweeps for (a) cc100, (d) cc82, (g) cc66, and (j) cc50 at different salt concentrations. Closed symbols represent the storage modulus *G*′, and open symbols represent the loss modulus *G*′′. Panels (b), (e), (h), and (k) are Van Gurp–Palmen plots that confirm the validity of a time–salt superposition (TSS). Panels (c), (f), (i), and (l) are the TSS curves obtained after shifting the frequency sweep data.

The viscoelastic properties of complex coacervates are known to be highly dependent on their water content and also on the strength and dynamics of electrostatic interactions. It is then very appropriate to highlight the effect of altering the charge density of the polyanion on the ability of the material to flow through linear rheology measurements. Consequently, small amplitude oscillatory shear experiments were performed at varying salt concentrations and charge densities of the P(SPMA_*x*_-*co*-OEGMA_*y*_) polyanions; these results are presented in Figure 4a,d,g,j. First, we observed that lower moduli values were measured for all complex coacervates when increasing the salt concentration, irrespective of the charge density of the polyanion. A similar decrease in moduli was found when the charge density of the polyanion was decreased from 100 to 50% at a fixed salt concentration. These observations are consistent with the results of the previous section, where we observed that an increase in the salt concentration or of the hydrophilic content in the polyanion leads to a lower polymer concentration in the complex. Huang et al. reported similar results when the charge density of both of the polyelectrolytes was decreased from 100 to 64% by incorporation of charge-neutral hydrophilic acrylamide units at 0.2 M NaCl.^25^ However, measurements were performed only for this single salt concentration, and the combined effects of both charge density and salt concentration were not investigated. Additionally, irrespective of the charge density of the polyanion, an increase in the salt concentration was shown to speed up the relaxation dynamics of the resulting complex coacervates. Usually, in the case that the relaxation dynamics of the complex coacervates are controlled by the salt concentration, it is common to superimpose the frequency sweep data obtained at various salt concentrations onto a single master curve.^15^ Here, the so-called TSS was demonstrated to be possible. First, satisfactory overlap of the phase angle δ as a function of the complex modulus |G*| was confirmed through van Gurp-Palmen plots for every series of complex coacervates formulated with different charge densities of the polyanion (Figure 4b,e,h,k).^36−38^ This is a frequency-independent plot, which is powerful for checking rheological complexities in polymeric systems. It eliminates the need to shift the original curves along the frequency axis and is supposed to yield salt concentration overlapped curves in case the TSS holds. Furthermore, it allows for direct determination of the residual vertical shift factor, *b*_s_, reported here (and in Figure S7) in the *x*-axis as a multiplication factor of the complex modulus |G*|. Then, for each series, the frequency sweep data were horizontally and vertically shifted compared to the samples prepared at 0.25 M NaCl, which were taken as references (Figure 4c,f,i,l). The horizontal and vertical shift factors (*a*_s_ and *b*_s_, respectively) used to superimpose the curves are reported in Figure S7. We can observe that the frequency range, where *G*′ ∼ *G*″, indicating Rouse dynamics where sticky points in the form of electrostatic interactions slow down the dynamics of the chains,^47,48^ shortens and eventually disappears while decreasing the charge density of the polyanion in the complex coacervates. For the series cc50, only the terminal regime characterized by slopes of 2 and 1 for *G*′ and *G*″, respectively, could be probed.^7,49^ It is worth noting that a slight deviation from the slopes of the terminal regime at the lowest frequencies investigated is visible for all the samples prepared at 0.0 M added NaCl. This is probably due to a kinetic trapping effect caused by the fast electrostatic complexation at such a low salt concentration upon mixing the oppositely charged polyelectrolytes. The possibility of performing the TSS for each of the coacervates formed with varying charge density of the polyanion indicates that the inclusion of the OEGMA hydrophilic units does not impact the mechanism by which the material relaxes but only the time scale at which it occurs, in a similar fashion as reported for a growing number of complex coacervate systems.^15,22,49−55^

**Figure 5:**
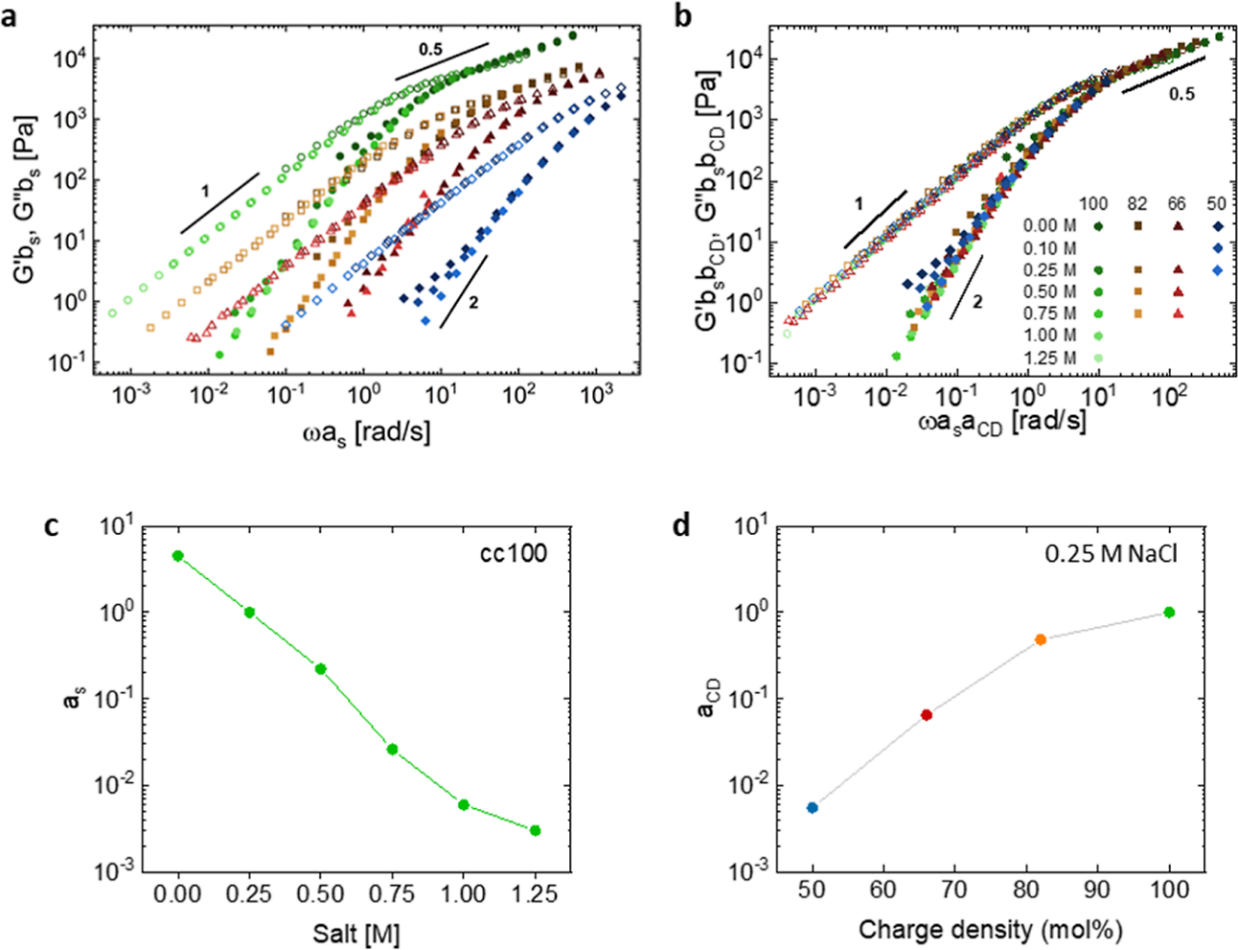
TSS curves for each charge density series (a) before and (b) after performing the time-salt-charge density superposition (TSCDS), where lower charge densities were shifted with regard to the series of reference cc100. (c) Horizontal shift factor *a*_s_ is a function of the NaCl concentration for the cc100 series. The sample prepared at 0.25 M NaCl is taken as the reference. (d) Horizontal shift factor, *a*_CD_, as a function of the charge density of the P(SPMA_*x*_-*co*-OEGMA_*y*_) polyanion after performing the TSCDS for the samples prepared at 0.25 M NaCl. The sample prepared with fully charged polymers (cc100) is taken as the reference.

Furthermore, upon plotting the TSS master curves for each of the four series of complex coacervates with varying charge densities of the polyanion, we can observe that they do not overlap (Figure 5a). More precisely, it appears that the relaxation dynamics become faster (i.e., the curves are shifted toward higher frequencies) when the charge density decreases. Following the same approach as for the TSS presented above, we are able to successfully overlap the four TSS curves onto a super master curve by performing a so-called TSCDS (Figures 5b and S8). The horizontal and vertical shift factors (*a*_CD_ and *b*_CD_, respectively) used to superimpose the curves are reported in Figure S9. This successful superposition highlights again that the addition of charge-neutral hydrophilic units causes only dilution of the electrostatic stickers between oppositely charged chains without affecting the relaxation mechanisms at play in the material. Finally, Figure 5c,d shows the evolution of the horizontal shift factors, *a*_s_ and *a*_CD_, respectively, for the cc100 series as a function of the salt concentration (the sample prepared at 0.25 M NaCl is taken as the reference) and for the series of complex coacervates prepared at 0.25 M NaCl with varying charge density of the polyanion (the fully charged sample, cc100, is taken as the reference). A decrease in the charge density by 50% of P(SPMA_*x*_-*co*-OEGMA_*y*_) speeds up the relaxation dynamics of the complex coacervate by almost 3 orders of magnitude (*a*_CD,50 mol %_ = 4.5 × 10^–3^), which is equivalent to an increase in the salt concentration from 0.25 to 1.25 M NaCl for the complex coacervates prepared with the fully charged PSPMA (*a*_s,1.25M_ = 2.5 × 10^–3^). This TSCDS principle for complex coacervate systems has already been reported for studies based on systems where at least one of the polymers is a weak polyelectrolyte, e.g., poly(acrylic acid) (PAA) or chitosan, for which the charged density could be changed by adjusting the pH.^52,56,57^ Similar to our work, for a certain range of pH values where the charge density of one of the polymers was moderately affected, the authors pointed out a speedup of the dynamics while decreasing the charge density without affecting the relaxation mechanisms. However, when critical pH values were exceeded (below pH 4.5 for PAA and above pH 6 for chitosan), the dynamics were shown to become independent of the charge density. The formation of dynamically arrested domains relying on H-bond association, which further delayed the relaxation dynamics, was hypothesized. Furthermore, in our recent study, for which a controlled amount of uncharged self-associative hydrophobic units were randomly included within the backbone of the polyanion chains, we showed that increasing the ratio of the hydrophobic groups led to gradual slowdown of the dynamics and a shift to lower frequencies.^22,55^

Overall, by putting our work in the perspective of these previous studies, we are able to highlight the efficiency of diluting the charges along one of the polyelectrolyte chains using charge-neutral, nonassociative water-soluble OEGMA units. This enables us to speed up the relaxation dynamics of the formulated complex coacervates while keeping the salt concentration unchanged. As stated in the Introduction section, the determination of the viscoelastic properties of the prepared complex coacervates provides valuable information when it comes to the design of injectable adhesives. The optimal balance between viscous and elastic behavior, respectively, affording injectability and resistance to rupture upon debonding, can therefore be primarily identified. Nevertheless, connecting linear viscoelastic rheology to the adhesion performance is useful only up to a certain degree; it is generally accepted that one needs to probe the nonlinear regime in order to understand the adhesion performance.

### 3.4 Adhesion Performance and Injectability of the P(SPMA_*x*_-*co*-OEGMA_*y*_)/QP4VP Complex Coacervates

**Figure 6:**
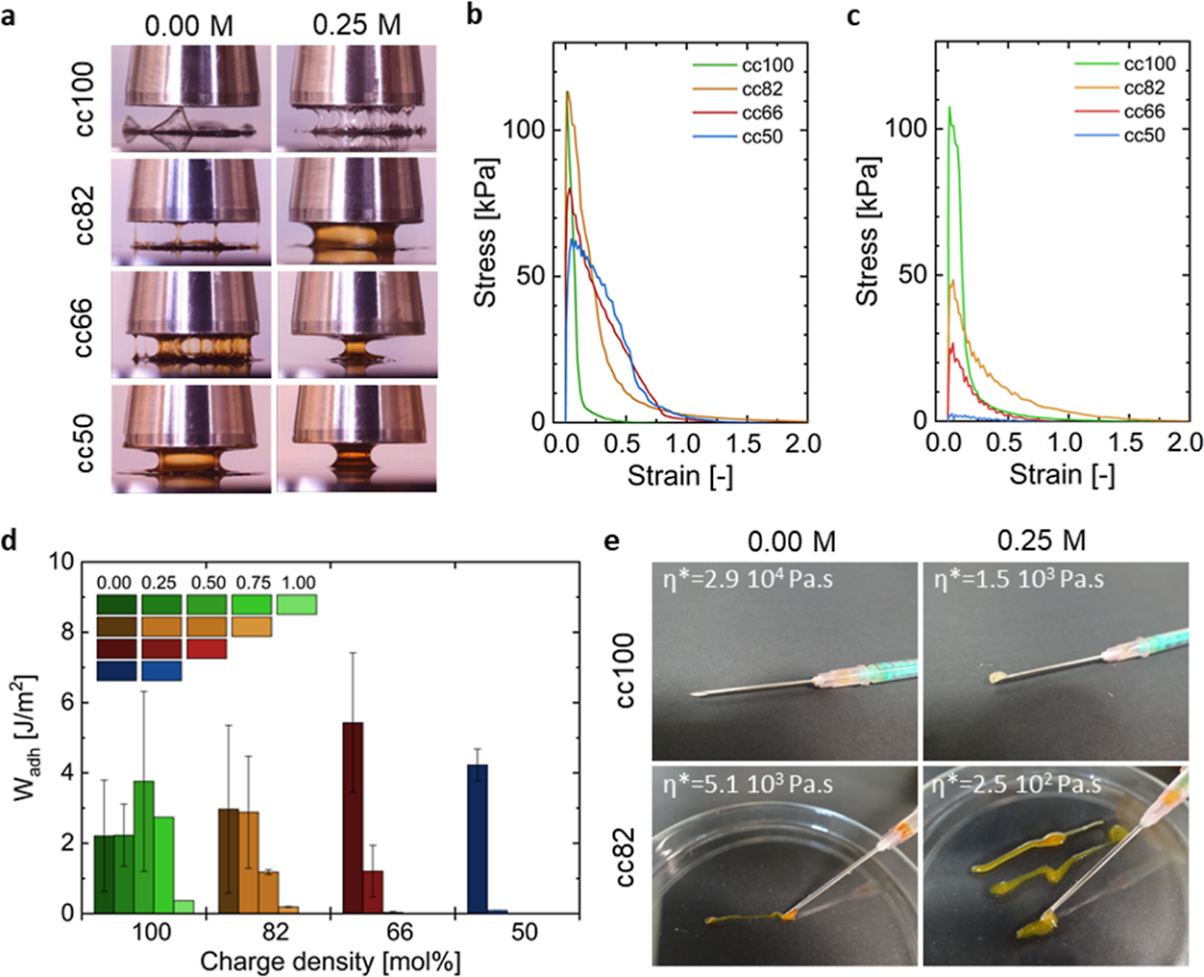
Probe-tack experiments. (a) Snapshots of the retraction of the probe for different conditions: from top to bottom cc100, cc82, cc66, and cc50; for 0.00 M NaCl in the left column and 0.25 M NaCl in the right column. Representative stress–strain curves for (b) 0.00 M NaCl and (c) 0.25 M NaCl. (d) Work of adhesion for different combinations of charge density and salt concentrations. (e) Injectability of the cc100 and cc82 complex coacervates at 0.00 and 0.25 M NaCl through a 1.2 mm diameter needle (G18). The complex viscosity measured at an angular frequency of 0.1 rad s^–1^ obtained from the frequency sweep data is reported in the top left corner of each picture. The injectability of the remaining samples is shown in Figure S10.

Probe-tack measurements were carried out to test the adhesion performance of our complex coacervates following procedures that have been successfully applied for similar systems.^7,12,22,55,58^ These nonlinear strain experiments usually provide valuable information about the mode of debonding (adhesive or cohesive failure), the force required to separate the two surfaces and the work of adhesion of PSAs.^9−11^ First, by visually looking at the debonding mechanism, adhesive failure was observed for cc100 and cc82 made with 0.00 M NaCl (Figure 6a and Movie S1). The corresponding stress–strain curves presented in Figure 6b,c also bear the signature of an adhesive debonding process, where a high peak stress is reached (above 100 kPa) in both cases before early detachment from the probe occurs. The ultimate strain before debonding barely reached 0.25. Correspondingly, the work of adhesion, *W*_adh_, (i.e., the energy needed to detach the two surfaces) obtained by integrating the area below the curves remained relatively low, with average values between 2 and 3 J m^–2^ (Figure 6d). Either upon increasing the salt concentration or decreasing the charge density of the P(SPMA_*x*_-*co*-OEGMA_*y*_) chains, a higher extent of bulk deformation with apparent fibrils was observed before failure occurred. In that case, a more favorable balance between the elastic and viscous components of the complex coacervates led to a higher energy dissipation upon debonding. This viscoelastic behavior is the most prominent for the cc100 sample prepared at 0.25 M NaCl and the cc82 and cc66 prepared at 0.00 M NaCl, where either cohesive or adhesive failure occurred in a strain range between 0.5 and 1. For the cc66 sample prepared at 0.0 M NaCl, the *W*_adh_ reached a maximum value of 5.5 J m^–2^. Eventually, by further increasing the salt concentration above 0.25 M NaCl or decreasing the charge density of the P(SPMA_*x*_-*co*-OEGMA_*y*_) chains below 66%, a clear liquid-like behavior was observed for all the samples. Only a single fibril was quickly obtained during retraction, and further cohesive failure occurred, with material residues left on both surfaces. The measured peak stress values are far below 50 kPa and *W*_adh_ values below 1 J m^–2^. This transition from solid-like adhesive debonding to viscoelastic debonding with the formation of fibrils to liquid-like cohesive debonding is reminiscent of traditional PSAs for which the cross-linking density, or cohesiveness, is gradually decreased.^9,12^ In addition, similar to PSAs, we observe a maximum of *W*_adh_ in the regime of viscoelastic debonding where the bulk energy dissipation is optimized. In the case of complex coacervates, an identical transition has been reported and obtained by increasing the salt concentration at which the complex coacervates were prepared.^6,7,22,55^ In light of these results, the charge density of one of the polyelectrolytes appears to be an efficient parameter for tuning the adhesive behavior of complex coacervates. Nevertheless, it is worth noting that the maximum work of adhesion obtained here (around 5.5 J m^–2^) remains noticeably lower compared to our previous study,^22^ or to the studies performed by Vahdati et al.^7^ or Dompé et al.^6,14^ (around 16 J m^–2^) where polyelectrolytes with a higher charge density and/or polyelectrolytes modified with a substantial amount of strongly associating hydrophobic units are used.

Finally, the injectability of the complex coacervate adhesives was verified by extrusion through a needle with a 1.2 mm internal diameter (G18; Figures 6e and S10). This qualitative observation was linked to the complex viscosity η* measured at an angular frequency of 0.1 rad s^–1^ obtained from the frequency sweep data, which can reasonably be correlated to the zero-shear viscosity of each sample. While it was not possible to push the cc100 complex coacervate made at 0.00 M NaCl through the needle, with some gentle hand pressure, it could get through at 0.25 M salt. However, injectability for cc100 was the easiest starting from 0.50 M salt. When the charge density of P(SPMA_*x*_-*co*-OEGMA_*y*_) was decreased, the injectability also became easier at lower salt concentrations. This simple yet meaningful extrusion test provides good insights into the improved injectability upon decreasing the charge density of the polyanion that fully correlates with the viscoelastic data reported earlier.

## 4 Conclusions

In this study, we verified the feasibility of BSPMA and OEGMA copolymerization and produced a range of strong anionic/charge-neutral P(SPMA_*x*_-*co*-OEGMA_*y*_) copolymers by adjusting the monomer ratios. A higher fraction of OEGMA, a charge-neutral and hydrophilic monomer, reduces the charge density of the polyelectrolyte, yet preserves its solubility in aqueous media. The resulting complex coacervates obtained by mixing with QP4VP, a strong cationic polymer, were shown to have an increased water content and a decreased salt resistance with a decreasing charge density. Additionally, either by increasing the salt concentration or decreasing the charge density of the polyanion, faster dynamics were observed. Consequently, samples with polyelectrolyte chains with low charge density at low salt concentrations exhibited similar properties as samples prepared with high charge density and high salt concentrations. TSS and TSCDS were performed to show that only the time scales of relaxation were altered when the charge density of the polyanion was changed but not the relaxation mechanism. Finally, the wet adhesion properties of the complex coacervates were shown to be independently tuneable with the salt concentration or the charge density of the polyanion used. For low salt concentrations, a decrease in the charge density was even shown to be favorable for enhancing the dissipative properties of the material and subsequently improving its adhesive performance. In parallel, injectability tests showed that the complex coacervates prepared at low salt concentrations and with lower polyanion charge densities were also more easily injectable. The method presented here allows us to reconcile the trade-off between the processability of complex coacervates at low salt concentrations and good wet adhesive properties. Consequently, it could be used as an additional tuning parameter for the design of complex coacervate-based injectable medical adhesives.

## Supporting Information Available

Protocols for polyelectrolyte synthesis, polyelectrolyte characterization by ^1^H NMR and SEC, calculations for complex coacervate preparation, shift factors for the TSS and TSCDS, Van Gurp–Palmen plots for the TSCDS, and injectability of the complex coacervates (PDF)
Probe tack experiments of the P(SPMA_*x*_-*co*-OEGMA_*y*_)/QP4VP Complex Coacervates (AVI)

## Supplementary Material

ma3c02352_si_001.pdf

ma3c02352_si_002.avi
