# Supplementary material for: Effect of Polyelectrolyte Charge Density on the Linear Viscoelastic Behavior and Processing of Complex Coacervate Adhesives
Source: Macromolecules. 2024 Jan 1;57(2):652–63. doi: 10.1021/acs.macromol.3c02352 (PMC10810003; doi:10.1021/acs.macromol.3c02352)
Supplement: Supplementary file 1 — ma3c02352_si_001.pdf [file ma3c02352_si_001.pdf]

Supporting Information For

Effect of Polyelectrolyte Charge Density on the Linear Viscoelastic Behavior and Processing of Complex Coacervate Adhesives

Larissa van Westerveld[a], Théophile Pelras[a,b], Anton H. Hofman[a], Katja Loos[b], Marleen Kamperman[a,*], Julien Es Sayed[a,*]

[a]*Polymer Science, Zernike Institute for Advanced Materials, University of Groningen, Nijenborgh 4, 9747 AG Groningen, The Netherlands*

[b]*Macromolecular Chemistry and New Polymeric Materials, Zernike Institute for Advanced Materials, University of Groningen, Nijenborgh 4, 9747 AG Groningen, The Netherlands*

*E-mail: marleen.kamperman@rug.nl

Polyelectrolytes synthesis

<u>*Kinetic study of BSPMA homopolymerization*</u>

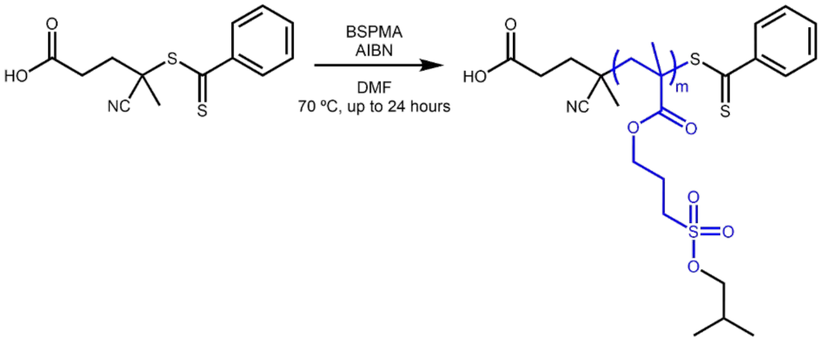

CTBPA (1 eq, 10.3 mg, 36.9 µmol), BSPMA (85 eq, 943 mg, 3.14 mmol), AIBN (0.1 eq, 0.591 mg, 3.60 µmol, 110 µL of a stock solution of 21.5 mg AIBN in 4.00 mL DMF) and 1.50 mL DMF were charged in a round bottom flask equipped with a stirring egg. An aliquot was withdrawn for ^{1}H NMR conversion analysis before the reaction mixture was deoxygenated via argon bubbling for 5 min. The vessel was immersed into a pre-heated oil bath at 70 °C and aliquots were withdrawn under argon protection at preset time intervals (*i.e.*, 1, 2, 4, 6, 8 and 24 hours). ^{1}H NMR samples (~ 2 drops) were directly diluted in $CDCl_3$ while SEC samples (~ 4 drops) were precipitated into cold 6:1 *n*-hexane:ethanol, dried in air and dissolved in SEC eluent. The remainder of the solution was discarded without further use.

Kinetic study of OEGMA homopolymerization

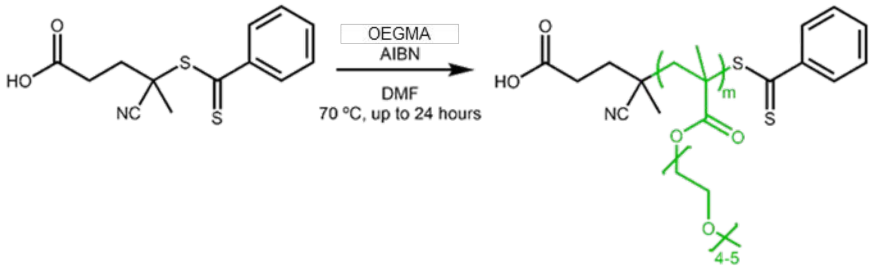

CTBPA (1 eq, 10.3 mg, 36.9 µmol), OEGMA (97 eq, 1.08 g, 3.61 mmol), AIBN (0.1 eq, 0.607 mg, 3.70 µmol, 113 µL of a stock solution of 21.5 mg AIBN in 4.00 mL DMF) and 1.50 mL DMF were charged in a round bottom flask equipped with a stirring egg. An aliquot was withdrawn for ^{1}H NMR conversion analysis before the reaction mixture was deoxygenated via argon bubbling for 5 min. The vessel was immersed into a pre-heated oil bath at 70 °C and aliquots were withdrawn under argon protection at preset time intervals (*i.e.*, 1, 2, 4, 6, 8 and 24 hours). ^{1}H NMR samples (~ 2 drops) were directly diluted in $CDCl_3$ while SEC samples (~ 4 drops) were precipitated into cold 6:1 *n*-hexane:ethanol, dried in air and dissolved in SEC eluent. The remainder of the solution was discarded without further use.

Kinetic study of BSPMA/OEGMA copolymerization

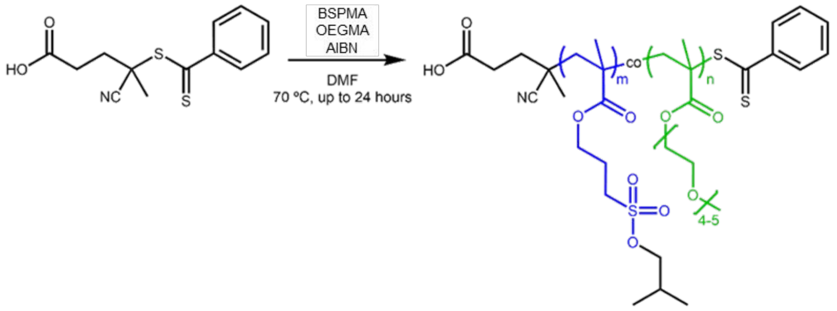

CTBPA (1 eq, 10.2 mg, 36.6 µmol), BSPMA (50 eq, 479 mg, 1.81 mmol), OEGMA (50 eq, 543 mg, 1.81 mmol), AIBN (0.1 eq, 0.640 mg, 3.90 µmol, 119 µL of a stock solution of 21.5 mg AIBN in 4.00 mL DMF) and 1.50 mL DMF were charged in a glass vial and mixed thoroughly. An aliquot was withdrawn for ^{1}H NMR conversion analysis before the reaction mixture was transferred into six 2 mL HPLC glass vials (~ 400 µL each) equipped with stirring bars and sealed with rubber septa. The vials were deoxygenated individually via argon bubbling for 3 min each before immersion into a pre-heated oil bath at 70 °C and were removed at set time points (*i.e.*, 1, 2, 4, 6, 8 and 24 hours). ^{1}H NMR conversion samples (~ 1 drop) were directly diluted in $CDCl_3$ and analysed. SEC samples (~ 4 drops) were precipitated into small volumes of cold 6:1 *n*-hexane:ethanol and dried in air before redissolving in eluent. The remainder of the aliquots (~ 350 µL) were precipitated into cold 6:1 *n*-hexane:ethanol, redissolved in THF and further precipitated into pure *n*-hexane before drying under high vacuum. ^{1}H NMR spectra of these purified samples were used to determine the relative conversion of BSPMA and OEGMA monomers by comparing the signal of BSPMA (2H, CH_2, 3.22 ppm) to that of OEGMA (3H, CH_3, 3.78 ppm and 2H, CH_2, 3.55 ppm).

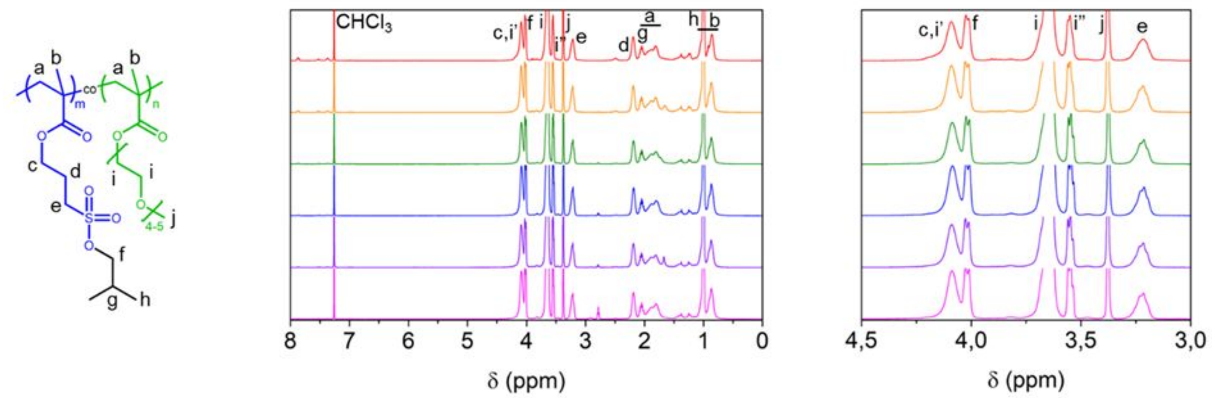

Figure S1. ^1H NMR ($CDCl_3$) analysis of purified aliquots withdrawn at different time points for a 50/50 copolymerization of BSPMA and OEGMA. Time points are: 1 h (red), 2 h (orange), 4 h (green), 6 h (blue), 8 h (purple) and 24 h (pink).

Synthesis of poly(oligo[ethylene glycol] methyl ether methacrylate) (POEGMA)

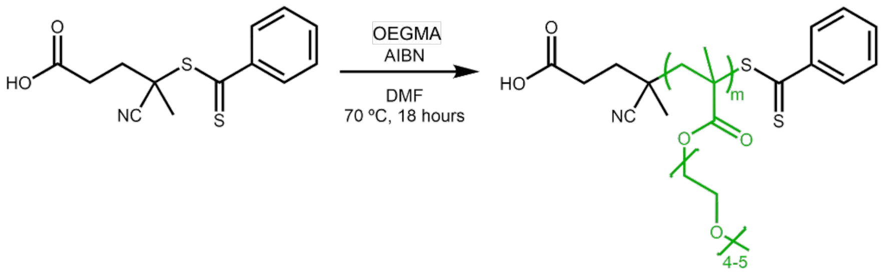

CTBPA (1 eq, 10.3 mg, 36.9 µmol), OEGMA (98 eq, 1.08 g, 3.60 mmol), AIBN (0.1 eq, 0.637 mg, 3.88 µmol, 118 µL of a stock solution of 21.5 mg AIBN in 4.00 mL DMF) and 1.5 mL DMF were charged in a round bottom flask equipped with a stirring egg. An aliquot was withdrawn for ^{1}H NMR conversion analysis before the reaction mixture was deoxygenated via argon bubbling for 5 min. The vessel was immersed into a pre-heated oil bath at 70 °C. After 18 hours, the vessel was cooled down to room temperature and opened to air before withdrawal of an aliquot for ^{1}H NMR conversion analysis. The polymer was precipitated into cold 6:1 n-hexane:ethanol, redissolved in minimal THF and further precipitated into pure n-hexane twice. The product was redissolved in minimal acetone, transferred into a glass vial and dried in vacuo. Yield: 916 mg. ^{1}H NMR: conversion = 87 %, DP = 85, $M_{n\,NMR}$ = 25 800 kg mol^{-1}. SEC: $M_{n\,SEC}$ = 24 200 kg mol^{-1}, Đ = 1.19.

Stability of POEGMA (i.e. negative control)

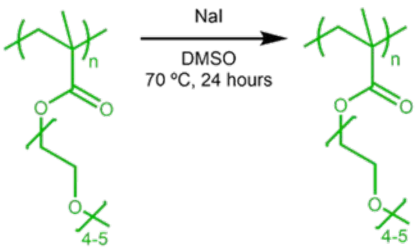

POEGMA85 (1 eq, 216 mg, 0.720 mmol OEGMA) and NaI (3 eq per OEGMA unit, 391 mg, 2.61 mmol) were dissolved in DMSO (5 mL, 40 mg per mL) and charged into a glass vial equipped with a stirring bar. The reaction mixture was left to stir at 70 °C for 24 hours. The resulting yellow solution was precipitated once in 2:1 n-hexane:ethanol, washed twice with 2:1 n-hexane:ethanol and once more with pure n-hexane. The polymer was redissolved in minimal acetone, transferred into a glass vial and dried in vacuo. Yield: 206 mg. SEC: $M_{n\ SEC}$ = 26 500 kg.mol^{-1}, Đ = 1.14.

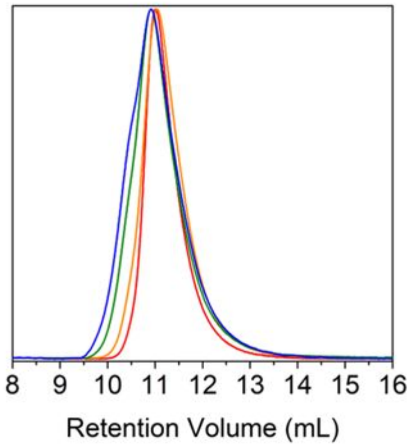

Figure S2. SEC elugrams of the P(BSPMA$_x$-*co*-OEGMA$_y$) protected intermediates measured in DMF with 0.01 M LiBr: x_{BPSMA} = 100 (red), x_{BPSMA} = 82 (orange), x_{BPSMA} = 66 (green) and x_{BPSMA} = 50 (blue).

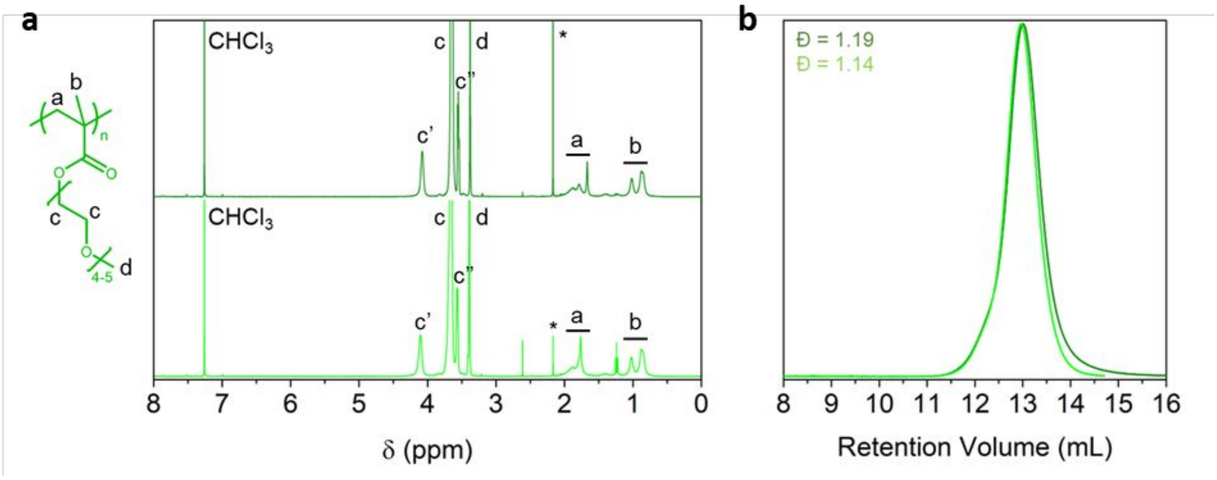

Figure S3. Stability test of POEGMA, where no differences in **(a)** ^{1}H NMR ($CDCl_3$) and **(b)** SEC trace can be observed.

Table S1: Compositions of the reaction mixtures for the synthesis of P($BSPMA_x$-*co*-$OEGMA_y$) copolymers.

	CTBPA		AIBN		BSPMA		OEGMA		DMF
	(mg)	(µmol)	(mg)	(µmol)	(g)	(mmol)	(g)	(mmol)	(mL)
BSPMA100*	106.0	378.0	6.20	38.0	12.0	45.4	0.00	0.00	17.0
BSPMA82	3.5	1.60	0.26	0.158	1.75	6.63	0.40	1.34	2.63
BSPMA66	4.5	2.05	0.34	0.250	1.80	6.84	1.04	3.48	3.38
BSPMA50	6.0	2.74	0.45	0.270	1.81	6.85	2.05	6.85	4.50

* The BSPMA100 has been synthesized in our previous study.[1]

Table S2: Compositions of the reaction mixtures for the synthesis of P(SPMA$_x$-*co*-OEGMA$_y$) copolymers.

	Copolymer			NaI		DMSO
	(mg)	(mg BSPMA)	(mmol BSPMA)	(mg)	(mmol)	(mL)
SPMA100	1500	1500	5.68	2560	17.1	30
SPMA82	1667	1349	5.10	2297	15.3	33
SPMA66	2556	1613	6.10	2748	18.3	51
SPMA50	3559	1666	6.30	2834	18.9	71

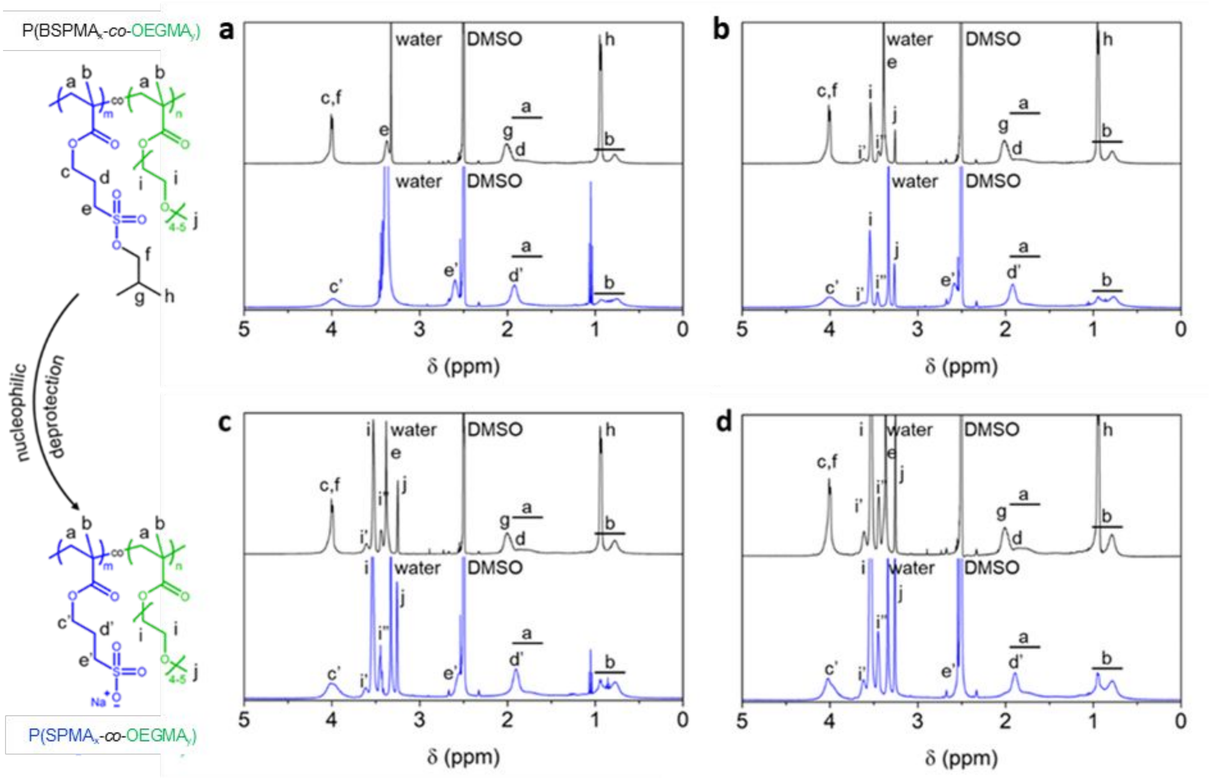

Figure S4. Comparative ^{1}H NMR spectra of the P(BSPMA$_x$-*co*-OEGMA$_y$) copolymer precursors (black) and their deprotected analogues P(SPMA$_x$-*co*-OEGMA$_y$) (blue) used in this study and measured in DMSO-*d$_6$*: **(a)** 100-0, **(b)** 82-18, **(c)** 66-34 and **(d)** 50-50. Traces of residual EtOH can be observed at 1.06 ppm and 3.44 ppm.

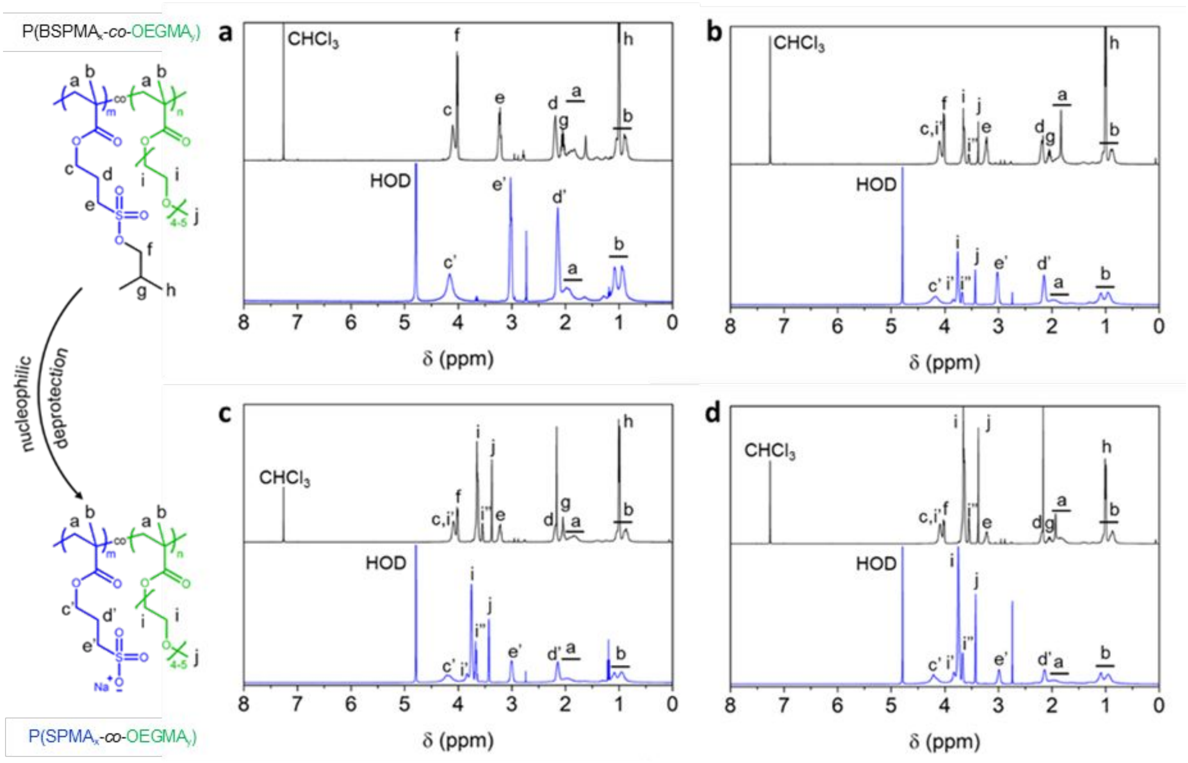

Figure S5. Comparative ^{1}H NMR spectra of the P(BSPMA$_x$-*co*-OEGMA$_y$) copolymer precursors ($CDCl_3$, black) and their deprotected analogues P(SPMA$_x$-*co*-OEGMA$_y$) (D_2O, blue) used in this study: **(a)** 100-0, **(b)** 82-18, **(c)** 66-34 and **(d)** 50-50.

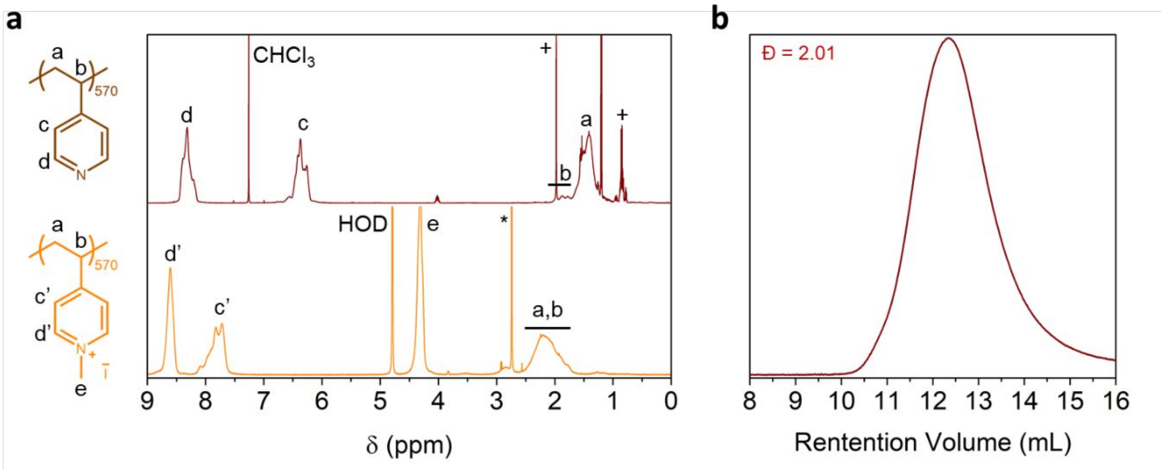

Figure S6. **(a)** Comparative ^{1}H NMR spectra of P4VP (brown, CDCl3) and QP4VP (orange, D_2O) and **(b)** SEC elugram (DMF with 0.01 M LiBr) of the commercially-available P4VP. +: residual ethyl acetate, *: residual DMSO.

Complex coacervates preparation

Table S3. Calculation of the amount of P($SPMA_x$-*co*-$OEGMA_y$) copolymers required for the preparation of final solutions with 0.05 M of charged SPMA units. For the complex coacervates, the amount of QP4VP was kept constant (0.25 mmol Q4VP, 61.8 mg)

V_{sample}	Final conc. [M]	Stock [M]	Volume [ml]	M SPMA [g mol^{-1}]	M OEGMA [g mol^{-1}]	Amount of SPMA [mmol]	Charge density [mol%]	Amount of OEGMA [mmol]	Weight of copolymer in coacervate [mg]
5	0.05	0.2	1.25	230	300	0.25	82	0.055	74.0
5	0.05	0.2	1.25	230	300	0.25	66	0.129	96.0
5	0.05	0.2	1.25	230	300	0.25	50	0.25	133.0

Rheology

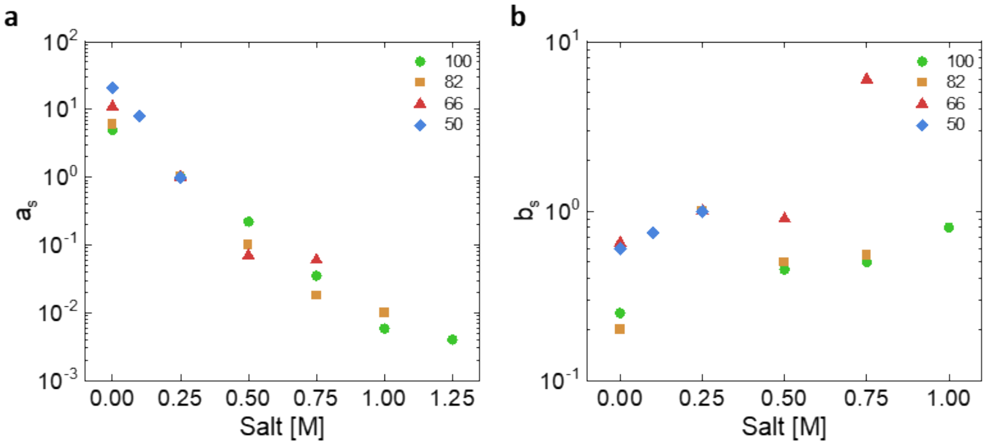

Figure S7. Shift factors used for the time-salt superposition of the complex coacervates: **(a)** horizontal shift factors a_s and **(b)** vertical shift factors b_s.

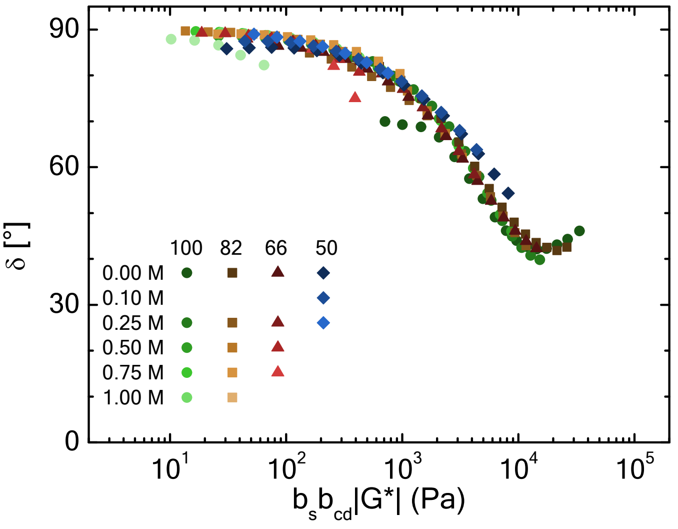

Figure S8. Van Gurp-Palmen plots confirming the possibility to perform the time-salt-charge density superposition of the complex coacervates formulated with P(SPMA$_x$-*co*-OEGMA$_y$) with varying charge density.

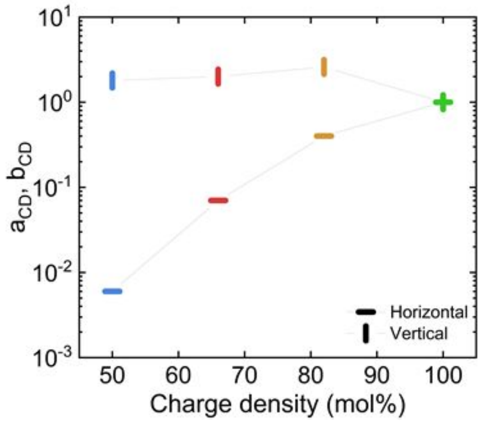

Figure S9. Horizontal (a_{CD}) and vertical (b_{CD}) shift factors used for the time-salt-charge density superposition of the complex coacervates formulated with P(SPMA$_x$-*co*-OEGMA$_y$) with varying charge density.

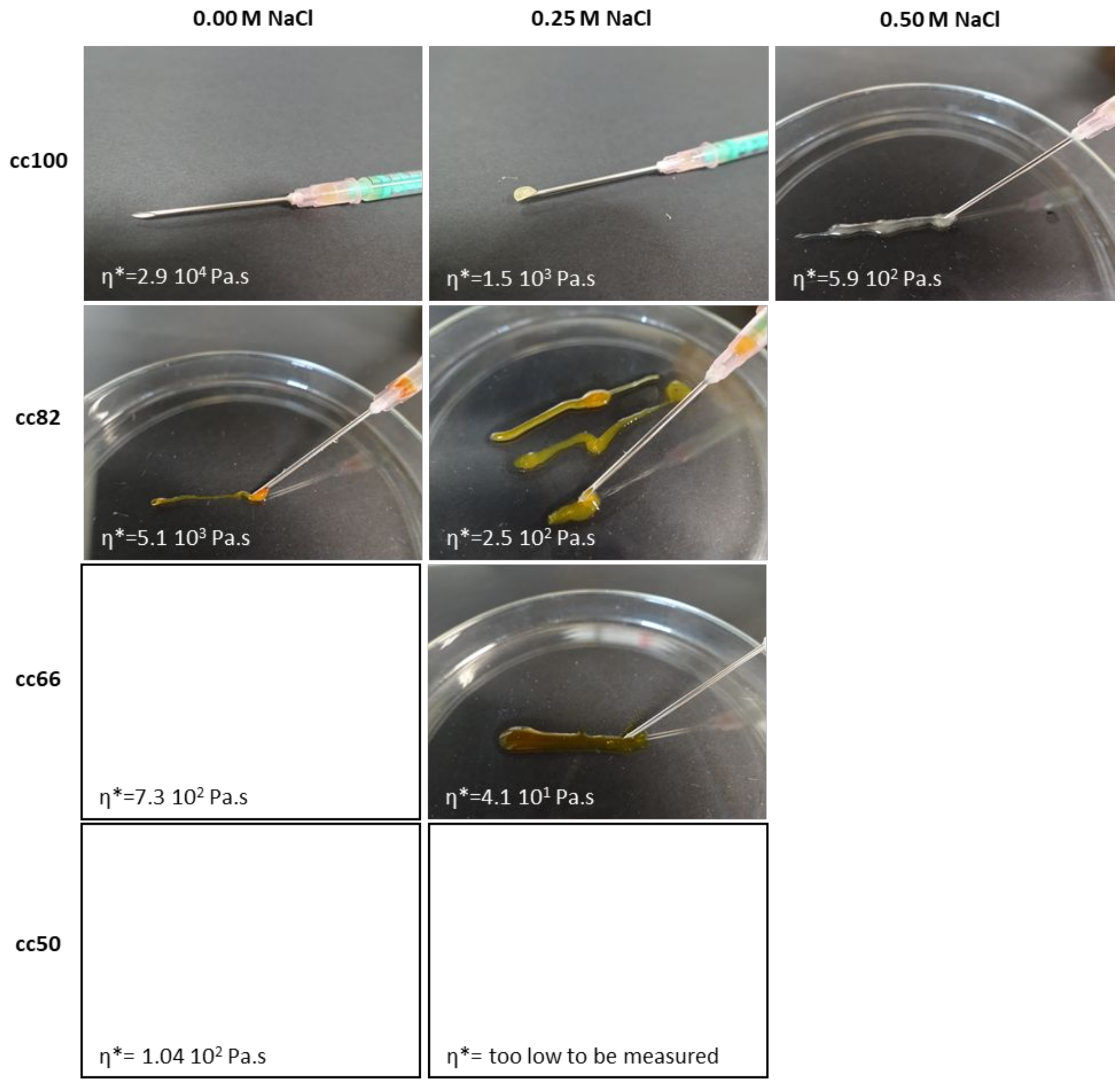

Figure S10. Injectability of the complex coacervates formulated with P($SPMA_x$-*co*-$OEGMA_y$) with varying charge density at 0.00 M NaCl, 0.25 M NaCl and 0.50 M NaCl through a 1.2 mm diameter needle (G18). The complex viscosity η* measured at an angular frequency of 0.1 rad s^{-1} obtained from the frequency sweep data is reported in the lower left corner of each picture. Unfortunately, not enough coacervate phase of cc66 at 0.00 M NaCl and cc50 at 0.00 M NaCl and 0.25 M NaCl was available to test their injectability.

Movie S1. Representative videos of the probe tack experiments performed at 0.00 M NaCl and 0.25 M NaCl for the cc100, cc82, cc66 and cc50 complex coacervates.

References

1. Hydrophobically modified complex coacervates for designing aqueous pressure-sensitive adhesives, *Soft Matter* **2023**, 19, 8832-8848.
